# Sensitive Troponins – Which Suits Better for Hemodialysis Patients? Associated Factors and Prediction of Mortality

**DOI:** 10.1371/journal.pone.0047610

**Published:** 2012-10-15

**Authors:** Ferruh Artunc, Christian Mueller, Tobias Breidthardt, Raphael Twerenbold, Andreas Peter, Claus Thamer, Peter Weyrich, Hans-Ulrich Haering, Bjoern Friedrich

**Affiliations:** 1 Department of Internal Medicine, Division of Endocrinology, Diabetology, Vascular Disease, Nephrology and Clinical Chemistry, University of Tuebingen, Germany; 2 Department of Internal Medicine, Division of Cardiology, University of Basel, Basel, Switzerland; 3 Dialysis Center, Leonberg, Germany; Max Delbrueck Center for Molecular Medicine, Germany

## Abstract

**Background:**

In hemodialysis patients, elevated plasma troponin concentrations are a common finding that has even increased with the advent of newly developed sensitive assays. However, the interpretation and relevance of this is still under debate.

**Methods:**

In this cross-sectional study, we analyzed plasma concentrations of sensitive troponin I (TnI) and troponin T (TnT) in stable ambulatory hemodialysis patients (n = 239) and investigated their associations with clinical factors and mortality.

**Results:**

In all of the enrolled patients, plasma TnI or TnT was detectable at a median concentration of 14 pg/ml (interquartile range: 7–29) using the Siemens TnI ultra assay and 49 pg/ml (31–74) using the Roche Elecsys high sensitive TnT assay. Markedly more patients exceeded the 99th percentile for TnT than for TnI (95% vs. 14%, p<0.0001). In a multivariate linear regression model, TnT was independently associated with age, gender, systolic dysfunction, time on dialysis, residual diuresis and systolic blood pressure, whereas TnI was independently associated with age, systolic dysfunction, pulse pressure, time on dialysis and duration of a HD session. During a follow-up period of nearly two years, TnT concentration above 38 pg/mL was associated with a 5-fold risk of death, whereas elevation of TnI had a gradual association to mortality.

**Conclusion:**

In hemodialysis patients, elevations of plasma troponin concentrations are explained by cardiac function and dialysis-related parameters, which contribute to cardiac strain. Both are highly predictive of increased risk of death.

## Introduction

Elevations of cardiac troponins in the plasma define are indicative of myocardial injury and necrosis [1]. According to the universal definition of myocardial infarction (MI) published in 2007, MI can be diagnosed based on a rise and fall in the plasma troponin concentrations above the 99^th^ percentile when there is evidence of myocardial ischemia [2]. However, in patients with end-stage renal failure (ESRD), chronic elevations at the subclinical level have been shown to occur without signs of myocardial ischemia [3]–[5]. Several longitudinal studies have consistently shown that elevated troponin concentrations in ESRD patients add prognostic information and are associated with increased mortality [6]–[10]. A meta-analysis concluded that increases in plasma troponin T or troponin I concentrations conferred a 2.64 or 1.74-fold increase, respectively, in mortality risk in ESRD patients [11]. The current view is that an elevated troponin concentration reflects chronic myocardial strain and damage during the course of renal failure and ESRD [12] rather than reduced clearance [13] or cross-reactivity with troponins from skeletal muscle [14], [15]. The cardiac work load of ESRD patients is greatly increased by several conditions, such as hypertension, shunt flow, chronic extracellular volume expansion, anemia or increased pulse pressure. These factors induce profound alterations in cardiac structure [16] and may lead to subsequent troponin release. In addition to a chronically increased cardiac work load, acute hemodialysis sessions induce cardiac stress due to circulatory alterations and may trigger myocardial stunning [17].

**Table 1:** Patient characteristics of the cohort (n = 239).

median age		70 (61; 77) years (n = 239)
gender distribution		64% male (n = 153)/36% female (n = 86)
renal disease	diabetic nephropathy	26% (n = 63)
	hypertension	8% (n = 19)
	glomerulonephritis	30% (n = 71)
	polycystic disease	5% (n = 11)
	other/unknown	31% (n = 75)
cardiac comorbidities	coronary heart disease	31% (n = 74)
	revascularized	19% (n = 31)
	valvular heart disease	26% (n = 61)
	atrial fibrillation	23% (n = 55)
	pulmonary hypertension	7% (n = 16)
	AICD carrier	2% (n = 4)
other comorbidities	diabetes mellitus	38% (n = 90)
	peripheral vascular disease	33% (n = 80)
	stroke	16% (n = 38)
	vasculitis	3% (n = 8)
	malignoma	14% (n = 34)
	COPD	8% (n = 19)
length of time on dialysis		46 (19; 85) months (n = 239)
duration of dialysis session		4.0 (4.0; 4.5) hours (n = 239)
dialysis access	arteriovenous fistula	71% (n = 169)
	PTFE graft	13% (n = 31)
	tunneled catheter	16% (n = 38)
dialysis membrane	high-flux	92% (n = 219)
	low-flux	8% (n = 20)
residual diuresis		250 (0; 1000) mL/day (n = 239)
anuric patients		39% (n = 93)
interdialytic weight gain		1.85 (1.29; 2.47) kg (n = 239)
blood pump speed		300 (280; 340) mL/min (n = 239)
shunt flow		1080 (733; 1475) mL/min (n = 187)
blood pressure		134 (122; 144)/69 (63; 74) mm Hg (n = 239)
pulse pressure		65 (54; 74) mm Hg (n = 239)
singe pool Kt/V		1.55 (1.40; 1.73) (n = 239)
laboratory data	hemoglobin	11.5 (11.1; 12.0) g/dL (n = 239)
	C-reactive protein	8.6 (4.6; 15.0) mg/L (n = 239)
	albumin	37.1 (35.4; 39.3) g/L (n = 239)
	parathormone	204 (130; 348) pg/mL (n = 239)
	β2-microglobulin	23.4 (19.4; 25.4) mg/L (n = 239)
LV systolic function	normal	59% (n = 140)
	mildly reduced	13% (n = 32)
	moderately or severely reduced	12% (n = 29)
	unknown	16% (n = 38)

Values shown are the median and interquartile range. N indicates number of patients from which data were available.

abbreviations:

PTFE  =  polytetrafluorethylene, LV  =  left ventricle, AICD  =  automated implantable cardioverter-defibrillator.

Typically, plasma troponin concentrations in ESRD patients are slightly elevated above the 99^th^ percentile touching the lower detection limit of many troponin assays employed in routine diagnostics. At this point, the analytical precision of conventional troponin measurements is reduced, and the coefficient of variation (CV) can reach 10–20% [18]. To overcome this problem, sensitive assays with a CV of <10% at the 99^th^ percentile have been developed and have shown improved accuracy in the diagnosis of MI [19]. Some of these tests can be designated as highly sensitive based on a high proportion (>50%) of subjects with measurable values that fall below the 99^th^ percentile [18], [20]. Currently, only few clinically available assays, such as the Roche Elecsys high sensitive (hs) TnT, fulfill this criterion [18]. There are only a few studies that have applied sensitive troponin assays in ESRD patients [21]–[23]. In one study, sensitive troponin T was found to be the most powerful predictor of mortality in ESRD patients among other established cardiac biomarkers [22].

**Figure 1:**
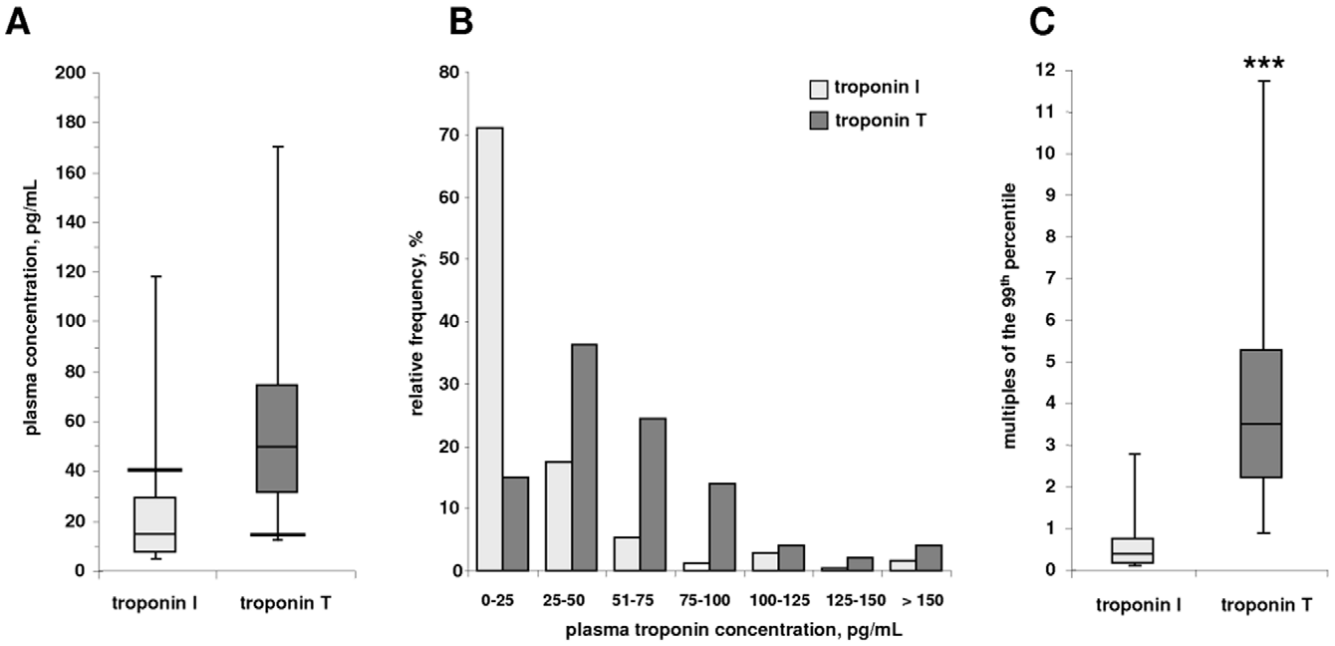
Depiction of the plasma troponin I and T concentrations in the cohort. **A** Box-and-Whiskers-Plot of the plasma troponin I and T concentrations indicating the median, interquartile range and the range between the 2.5^th^ and 97.5^th^ percentile. Solid lines represent the 99^th^ percentile of the assay [19]. **B** Distribution of the plasma troponin I and T concentrations in the cohort (n = 239). **C** Box-and-Whiskers-Plot of the plasma troponin I and T concentrations expressed as multiples of the 99^th^ percentile [19]. ^***^ indicates a significant difference (p<0.0001) between the troponin T and troponin I concentrations.

With the advent of sensitive assays, cardiac troponins are now detected to a larger extent in hemodialysis patients raising questions about interpretation and relevance to nephrologists and cardiologists alike. The increased sensitivity and resolution of the assays, however, also improve the chance to identify factors that are associated with elevated plasma troponin concentrations, such as loss of residual renal function [24] or volume overload [25], both of which contribute to cardiac strain and are predictive of increased mortality. Since both troponins (I and T) can be measured with sensitive assays, results might also reflect different biochemical properties. Therefore, we measured both troponin I and T with sensitive assays in a comparative approach to investigate associated factors and to analyze the diagnostic performance in predicting death during a follow-up period of two years.

**Table 2:** Univariate correlations (Pearson's r) of the plasma troponin concentration with general and dialysis-specific parameters (n = 210–239).

	troponin I, pg/ml	troponin T, pg/ml
age, y	0.32 ^***^	0.43 ^***^
systolic LVF, classes	0.44 ^***^	0.38 ^***^
time on dialysis, months	0.15 ^*^	0.18 ^**^
residual diuresis, ml/day	−0.13 ^*^	−0.29 ^***^
interdialytic weight gain, kg	0.13 ^*^	0.25 ^**^
shunt flow, ml/min	−0.16 ^*^	−0.14 #
systolic blood pressure, mm Hg	0.11 #	n.s.
diastolic blood pressure, mm Hg	−0.13 ^*^	−0.19 ^**^
pulse pressure, mm Hg	0.21 ^*^	0.22 ^**^
duration of a HD session	n.s.	n.s.
blood pump speed, ml/min	n.s.	0.15 ^*^
Kt/V	n.s.	n.s.
hemoglobin, g/dL	n.s.	n.s.
plasma albumin, g/L	n.s.	n.s.
C-reactive protein, mg/L	n.s.	0.21 ^**^
parathormone, pg/mL	0.16 ^*^	0.14 ^*^
β2-microglobulin, mg/dL	n.s	n.s

# p<0.10, ^*^ p<0.05, ^**^ p<0.01, ^***^ p<0.001, n.s.  =  not significant (p>0.10).

## Methods

### Patients and cohort

This cross-sectional, prospective multicenter study was conducted in stable, ambulatory hemodialysis patients from four dialysis centers in southwest Germany between September 2009 and April 2010. Patients without evidence of an acute illness and cardiac event or procedure within two months were included in the study after providing written informed consent. Patients with cardiac diseases leading to increased plasma troponin concentrations independent of ESRD, such as amyloidosis, were excluded. The study was approved by the local ethics committee of the University hospital Tuebingen.

**Figure 2:**
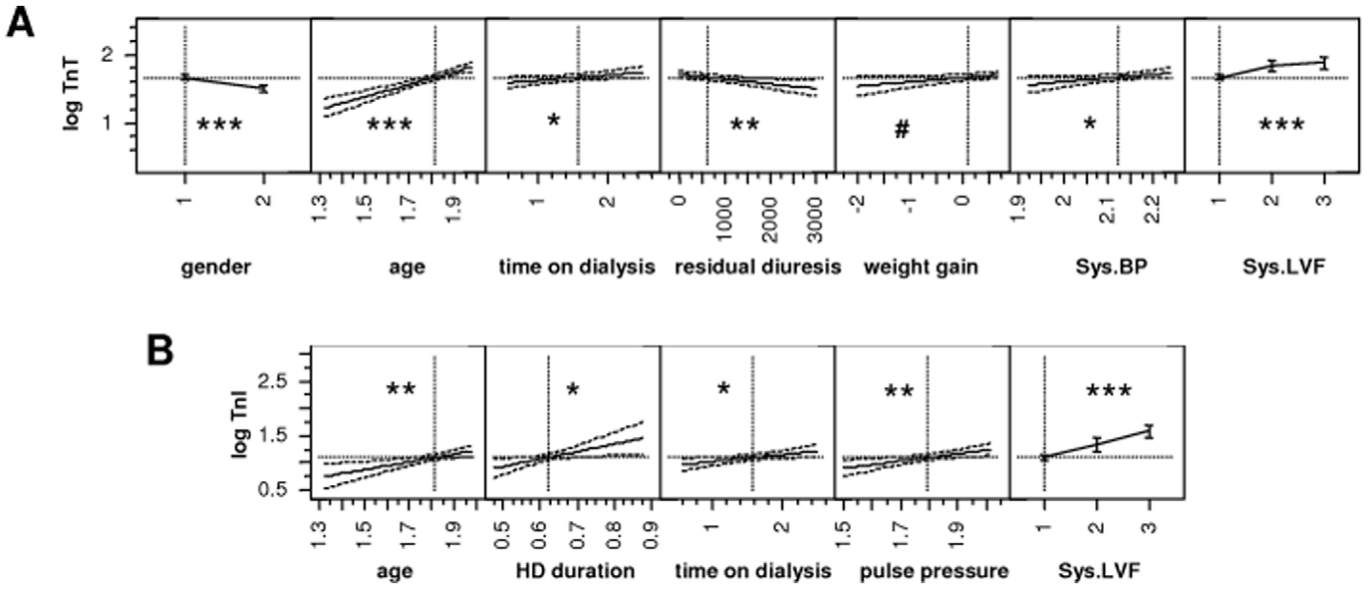
Plot showing the influence of single predictors in the multivariate model for troponin T (A) and troponin I (B). ^#^ p<0.10, ^*^ p<0.05, ^**^ p<0.01, ^***^ p<0.001.

**Figure 3:**
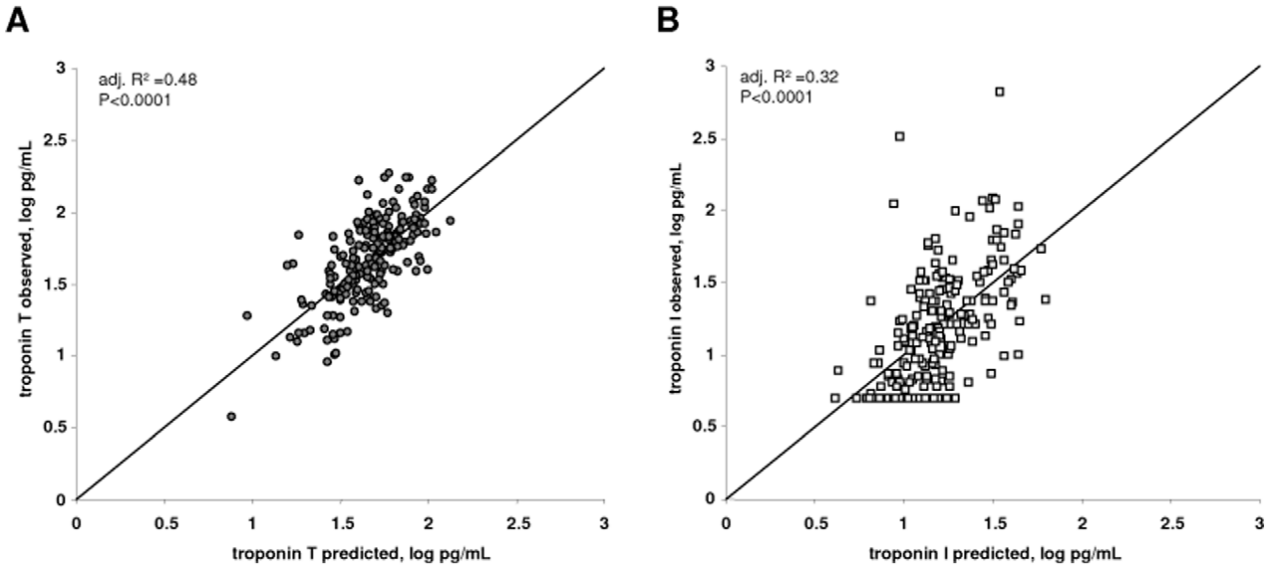
Agreement between predicted and observed log troponin I and T concentrations. Plot of the predicted and observed troponin values for troponin T (**A**) and troponin I (**B**) as a result of the models shown in table 4. The diagonal line represents the line of identity.

**Table 3:** Independent factors determining plasma troponin concentrations by multivariate linear modeling (n = 201).

	covariate	estimate ± SD	standardized estimate ± SD	p-value
**troponin T, pg/ml**	y-intercept	−1.25±0.61	1.59±0.02	0.0410
	age, y	0.84±0.14	0.27±0.05	<.0001
	systolic LVF, classes [2 vs. 1]	0.14±0.04	0.14±0.04	0.0016
	systolic blood pressure, mmHg	0.55±0.27	0.09±0.04	0.0432
	residual diuresis, ml/24 h	−6 10^−5^±2 10^−5^	-0.09±0.03	0.0050
	gender [1 = male]	0.08±0.02	0.08±0.02	<.0001
	interdialytic weight gain, kg	0.06±0.03	0.07±0.04	0.0688
	time on dialysis. months	0.08±0.04	0.07±0.04	0.0395
	systolic LVF, classes [3 vs. 2]	0.04±0.27	0.06±0.06	0.4715
	atrial fibrillatiom [1 = yes]	0.08±0.04	0.04±0.02	0.0324
**troponin I, pg/ml**	y-intercept	−2.32±0.69	1.10±0.03	0.0009
	duration of a HD session, h	1.30±0.58	0.26±0.12	0.0274
	systolic LVF, classes [3 vs. 2]	0.22±0.07	0.25±0.08	0.0042
	systolic LVF, classes [2 vs. 1]	0.25±0.08	0.22±0.07	0.0013
	age, y	0.62±0.24	0.20±0.08	0.0104
	pulse pressure, mm Hg	0.71±0.24	0.18±0.06	0.0032
	time on dialysis, months	0.11±0.06	0.11±0.05	0.0410
	valvular disease [1 = yes]	0.10±0.06	0.05±0.03	0.0767

### Laboratory assays

Plasma concentrations of troponin I and troponin T were measured in three independent samples collected within two weeks. Each sample was collected prior to the start of a dialysis session. Blood was collected in lithium-heparinized tubes (Sarstedt, Nuembrecht, Germany), cooled at 4°C, and centrifuged within 4 hours. The sera were stored at −80°C until further analysis. Plasma concentrations of troponin I were measured using the Troponin I Ultra assay on a Siemens ADVIA Centaur system (Siemens Healthcare Diagnostics, Eschborn, Germany) with a detection limit of 6 pg/ml, a 99^th^ percentile at 40 pg/ml, and a CV of less than 10% at 30 pg/ml, as specified by the manufacturer. Plasma concentrations of troponin T were measured using an automated Roche assay on an Elecsys 2010 system with a detection limit of 2 pg/ml, a 99^th^ percentile at 14 pg/ml, and a coefficient of variation of less than 10% at 13 pg/ml [19]. Plasma beta-2-microglobulin concentrations were measured using a turbidimetric assay (Randox Laboratories, Antrim, United Kingdom) on a Siemens ADVIA 1800 system with a measurable range between 0.56–20.9 mg/l and an upper reference concentration of 3 mg/l [26]. All other laboratory values (parathormone, hemoglobin, albumin and C-reactive protein) were extracted from the patients' medical records and represent an average of the available values from the previous year (4–12 values).

**Figure 4:**
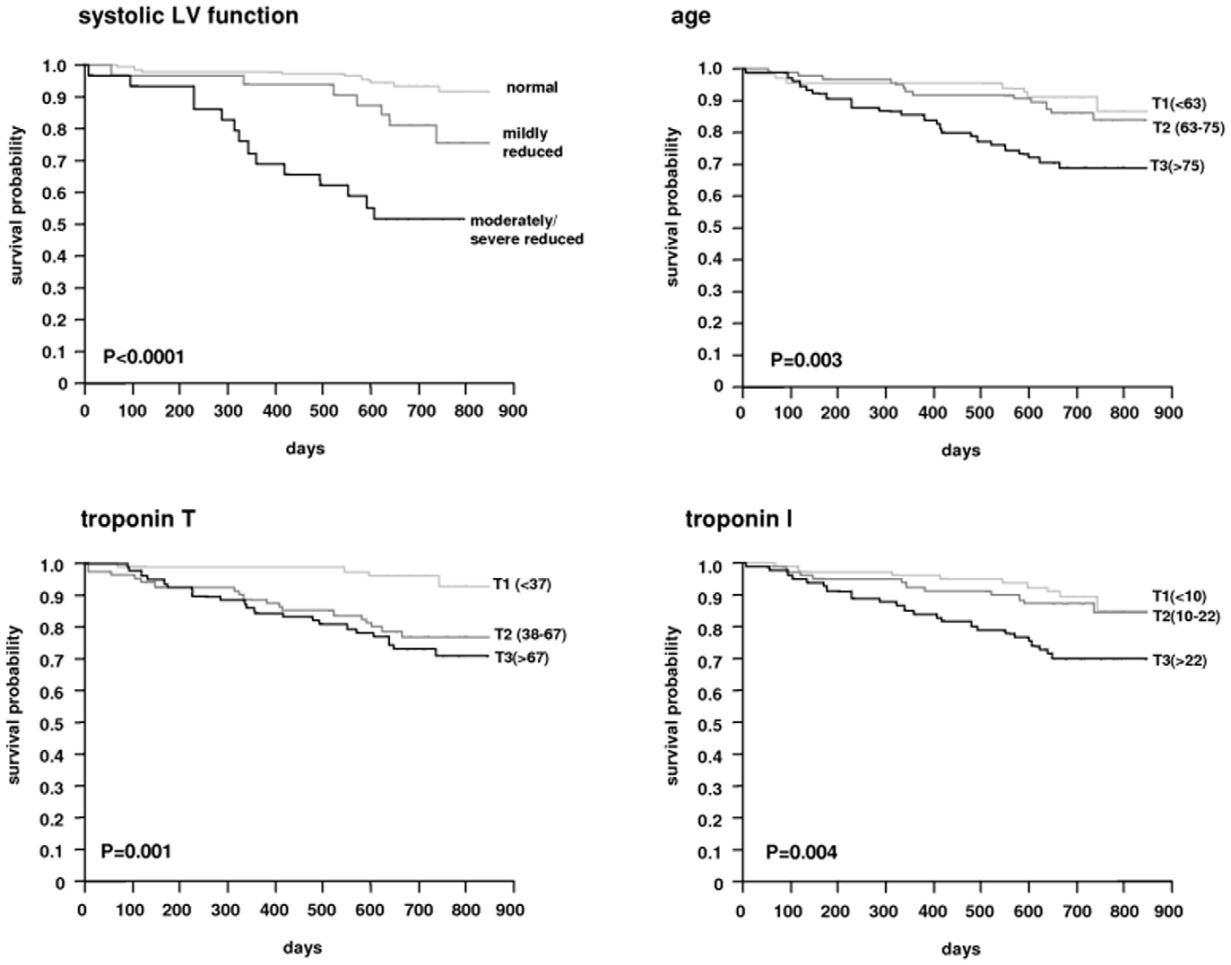
Survival curves of the cohort stratified according to classes of systolic LV function and tertiles of age, troponin T and troponin I.

**Figure 5:**
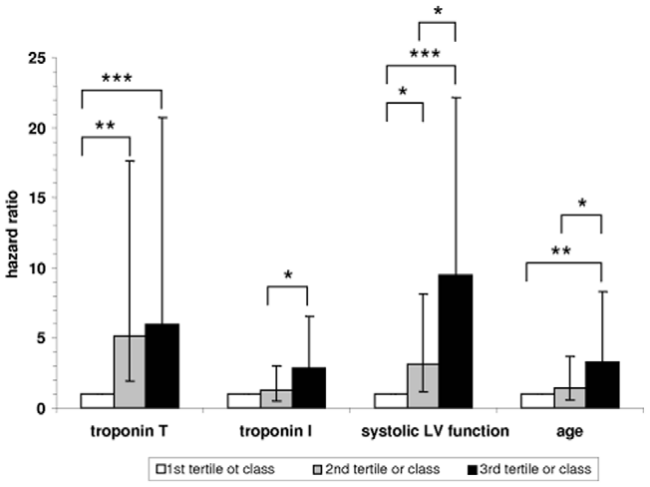
Hazard ratio of mortality according to tertiles of troponin T (<37; 38–67; >67 pg/ml), troponin I (<10; 10–22; >22 pg/ml), age (<63; 63–75; >75 years) and classes of systolic LV function (1 = normal, 2 = mildly reduced; 3 = moderately and severe reduced). Error bars represent lower and upper 95% confidence interval. The hazard ratio of the first tertile or class, respectively, was set to 1. ^**^ p<0.01, ^***^ p<0.001.

**Table 4:** Prognostic performance of the plasma troponin concentration in predicting death during follow-up.

	AUC (c-index)	cut-off, pg/ml	Youden index	sensitivity, %	specificity, %	positive predictive value, %	negative predictive value, %
troponin T	0.684	38	0.32	91	41	26	95
troponin I	0.665	21	0.32	61	70	16	77

### Clinical data

The following data were extracted from each patient: residual diuresis, as measured by 24 h urine collection: single pool Kt/V (mean of the most recent 4 values collected), interdialytic weight gain, predialytic systolic and diastolic blood pressure (mean of the most recent 12 values), dialysis access and membrane, length of time on dialysis, blood pump flow and shunt flow measured using a Transonic system (Ithaca, NY, USA). The left ventricular (LV) systolic function was classified from available echocardiography examinations in which class 1 indicated normal function, 2 was mildly reduced, and 3 was moderately or severely reduced. Determination of the LV systolic function was done at the discretion of the cardiologist and not standardized. Echocardiography was available in 84% of all patients within one year (plus-minus) relative to study enrollment.

### Statistical analysis

Three samples were available in 87% of the patients and were averaged to calculate the arithmetic mean without excluding possible outliers. For further analysis, plasma troponin concentrations and continuous clinical data were log transformed to approximate normal distribution. The association of the plasma troponin concentrations with clinical or dialysis-related factors was analyzed by univariate parametric correlation. Multivariate linear regression analyses were performed to identify independent determinants of the plasma troponin concentrations. Selection of the variables entering the model were derived from forward, stepwise multiple linear regression, and all variables with a p-value <0.05 were included in the multivariate linear regression models. The residuals of each model were tested for normality. Averaged values of the deceased patients were compared to those from the surviving patients using t-Test or Wilcoxon's test. Kaplan-Meier curves were generated after stratification into tertiles of the variable according to its distribution. The follow-up period started on the first day of blood draw and was censored as of 31 December 2011. The diagnostic performance was analyzed using receiver-operator curves (ROC or c-index) and the best cut-off value was considered as the maximal difference of sensitivity and 1-specificity (Youden index). C-Index was calculated using Wessa, P. (2012), Free Statistics Software, Office for Research Development and Education, version 1.1.23-r7, http://www.wessa.net/. Univariate and multivariate proportional hazards were calculated to analyze the risk ratios of each predictor and the independence of the predictors. Data analysis was performed using the statistical software package JMP 10.0.1 (SAS Institute, Cary, NC).

## Results

### Patients

Of the n = 250 available patients that were treated in the participating centers, n = 239 were included in the study. The reasons for exclusion of the n = 11 patients were the following: patients declined to participate (n = 6), deaths occurred following the initial plasma collection (n = 2), cardiac amyloidosis (n = 2) and a recent enrollment in dialysis (n = 1). The characteristics of the study cohort are provided in table 1. Participants were primarily geriatric, with a median age of 70. They had been in a dialysis-dependent state for a median of 45 months and were dialyzed mainly using an arteriovenous (AV) fistula (71%) or a high flux membrane (95%). The dialysis sessions, which lasted 4 hours, had a Kt/V value above the recommended K/DOQI target value of 1.3. Median shunt flow was approximately 1 L/min. Residual diuresis was low (median of 250 ml/day), and 39% of the patients were anuric. The median plasma albumin concentration was slightly below the recommended KDOQI target of 40 g/L, although hemoglobin and parathormone levels were within the target range. Plasma β2-microglobulin concentrations were highly elevated (median concentration of 23.5 mg/L), indicating an accumulation due to reduced renal and low dialysis-driven clearance.

Cardiac comorbidities were present in a large proportion of the patients. Ischemic heart disease was the most frequent condition (31%). Systolic LV dysfunction was found in 26% of all of the patients (table 1).

### Plasma troponin concentrations in the cohort

Plasma troponin I or T was detectable with the applied sensitive assays in all of the enrolled patients. The median TnT and TnI concentrations were 49 pg/ml and 14 pg/ml, with an interquartile range of 31 to 73 pg/ml and 7 to 28 pg/ml, respectively (Fig. 1A). The distributions of the plasma troponin concentrations are shown in Figure 1B. A large proportion of patients (95%) had a plasma TnT concentration above the 99^th^ percentile at 14 pg/ml, while only 14% of the patients had a plasma TnI concentration above the 99^th^ percentile at 40 pg/ml. Expressed as multiples of the 99^th^ percentile, the TnT concentration was elevated 3.5 fold (interquartile range: 2.2 to 5.2) compared to 0.4 fold for TnI (interquartile range: 0.2 to 0.7; Figure 1C), which represented a highly significant difference (p<0.0001). Plasma troponin concentrations were moderately correlated with each other (r = 0.63, p<0.0001). TnI had a greater variability within the three samples with a variation of 26±24% compared to 8±8% for TnT.

### Univariate analyses

Table 2 lists the results of univariate correlation analyses of the plasma TnI and TnT concentrations with the collected parameters. Both troponins showed a significant positive correlation with age, degree of systolic dysfunction, interdialytic weight gain, pulse pressure and parathormone concentration. The troponins were negatively correlated with residual diuresis, diastolic blood pressure and shunt flow. TnT was positively correlated with blood pump speed and C-reactive protein concentrations. There was no correlation with the plasma β2-microglobulin concentration. Variations in TnT and TnI, expressed as the standard deviation of the three samples obtained from each patient, were not correlated to any of the parameters studied.

### Multivariate analyses

To analyze independent determinants of the plasma TnI and TnT concentrations, multivariate linear regression modeling was performed. The parameters that entered the final model were selected using a stepwise forward approach with a p-value of <0.05. Table 3 and Figure 2 show the results of the multivariate linear regression models that were applied. Besides age and gender, the independent determinants of the plasma TnT concentration were systolic LV function, systolic blood pressure, time on dialysis and residual diuresis. The influence of interdialytic weight gain was close to reaching statistical significance (p = 0.0688). Overall, the adjusted r^2^ of the model was 0.48, indicating that 48% of the variability in plasma TnT concentrations could be explained by these factors (Figure 3A). In contrast, the best model for TnI had an adjusted r^2^ of 0.32, and age, systolic LV function, pulse pressure, length of time on dialysis and duration of a HD session were independent predictors (Figure 3B).

### Prognostic value

After the initial blood draw, the cohort was followed for survival to analyze the prognostic value of elevated plasma troponin concentrations. During a median follow-up time of 710 days (679; 761), 44 patients died corresponding to an annual mortality rate of 9.2%. Compared to surviving patients, deceased patients were significantly older (77 years [68;82] vs. 69 [59; 76]; p<0.0001), were more likely to have LV dysfunction (68% vs. 24%; p<0.0001) and had lower diastolic blood pressure (65 mm Hg [59;72] vs. 69 [63; 74]; p = 0.0087). They had higher plasma CRP and parathormone concentrations (12.9 mg/L [8.8;20.1] vs. 7.7 [4.2; 13.6]; p<0.0001 and 240 pg/mL [169;431] vs. 203 [124; 326]; p = 0.0011, respectively). Plasma troponin T and I concentrations were also significantly (p<0.0001) higher in deceased patients (67 [46;87] pg/mL and 24 [11;46] pg/mL, respectively) compared to surviving patients (44 [29;70] pg/mL and 13 [7]; [24] pg/mL, respectively).

The survival curves stratified for tertiles of age, plasma troponin T and I concentrations as well as classes of systolic LV function are shown in Figure 4. The relative risk compared to the first tertile or class is shown in Figure 5. Systolic LV function was the strongest predictor of death during follow-up, followed by increased plasma troponin T concentration. When analyzing the diagnostic performance of plasma troponin T and I concentrations using contingency tables and receiver-operator curves (Table 4), both troponins showed similar AUC-values, however, plasma troponin T concentration had a higher sensitivity (91%) and negative predictive value (95%) compared to troponin I (61% and 77%, respectively). For each increase of the plasma troponin T or I concentration by 10 pg/ml, the risk ratio was increased by 13.9% and 5.2%, respectively. In a multivariate proportional hazards model with age, systolic function and troponin concentration, only systolic function and age remained independent predictors (data not shown).

## Discussion

This study shows that plasma troponin T concentrations measured using a highly sensitive assay were elevated in the vast majority of the hemodialysis cohort studied and that both cardiac and dialysis-related parameters determined its concentrations. Compared to sensitive troponin I, TnT was elevated in more patients, reflected dialysis-related factors to a greater extent and tended to be a stronger predictor of mortality. This study is in agreement with previous studies of patients without ESRD that showed an association between increased plasma troponin concentrations and impaired systolic function [27], [28]. In addition, plasma troponin concentrations were independently associated with dialysis-related parameters, such as time on dialysis, residual diuresis, interdialytic weight gain or duration of a HD session. These factors, which can be termed “dialysis heritage”, are strongly interrelated to each other in that residual function was lost with an increasing time on dialysis, which favors volume overload. Residual function and interdialytic weight gain are both independent risk factors for increased mortality in ESRD patients [24], [25]. The association between residual diuresis and plasma troponin concentration has been reported earlier [29].

Most of the studies evaluating plasma troponin concentrations in ESRD patients have investigated the prognostic value and association with mortality. However, only a few studies have addressed the determinants of plasma troponin concentration in ESRD patients. Several studies found that elevated troponin concentrations were associated with the extent of coronary disease [30], [31], calcification [32] or left ventricular hypertrophy or dysfunction [33]. Interestingly, in our study shunt flow was negatively correlated with plasma troponin T or I concentrations and was an independent predictor with a borderline statistical significance in the multivariate analysis (p = 0.07). Despite the possible detrimental effects of a high shunt flow on the heart [16], our results suggest that a high shunt flow can be interpreted as a sign of better cardiac status as judged by a lower plasma troponin concentration. This also highlights the fact that association studies cannot distinguish between cause and consequence and can be influenced by phenomena such as reverse causation (i.e., a failing heart that leads to increased plasma troponin concentrations and low shunt flow).

In studies comparing troponin T to troponin I, ESRD patients were 2–3 times more likely to have elevated concentrations of troponin T than troponin I [34], [35]. In this study, TnT was elevated in nearly all of the patients (95%) compared to TnI (14%). This is similar to the findings of Jacobs et al., who found elevations of TnT measured with the high sensitive Roche assay in all of the patients studied whereas TnI measured using a conventional assay was elevated in only 28% of the cases [21]. Using a sensitive troponin I assay and a cut-off point of 35 pg/ml, this proportion was increased to 37% [23]. This difference can be partially explained with our data, which showed a slight accumulation of troponin T during the course of ESRD in that residual diuresis and the length of time on dialysis were independent predictors of the plasma concentration of only TnT, but not TnI. The finding that plasma troponin I concentrations were not influenced by residual renal clearance is in agreement with a study in ESRD patients that showed no differences in the clearance of troponin I after an acute MI [36].

However, there should be caution when interpreting high troponin T values as mere accumulation [5], [37]. Elevated troponin T and troponin I concentrations are both strong predictors of mortality. Accumulation of troponin T can only occur following release after myocyte damage and should be regarded as a pathologic finding in any ESRD patients. Furthermore, accumulation can only occur when residual renal function declines, which is a strong predictor of survival in hemodialysis patients [38]. Therefore, plasma troponin T concentrations provide information about cardiac release and dialysis heritage, which might explain the improved prognostic value of troponin T compared to troponin I, as seen in our study or in the study of Jacobs et al. [18], [20].

A more meaningful cut-off point than the 99^th^ percentile would be levels that are linked to an increased mortality risk. We found that a plasma troponin T concentration above 38 pg/mL which is roughly three-times the 99^th^ percentile was associated with a 5-fold risk of death during 2 years of follow-up. In contrast, we could not find a clear cut-off for troponin I as the association with death was more gradual. So far, only one study reported the association of hs TnT with mortality and found reduced survival rates at levels above 24.15 pg/ml [22]. In our study, both troponins were not independent predictors of death such as age or systolic LV function and did not add prognostic information. However, they can be used as alternative markers to identify patients at risk when echocardiography or other cardiac workup is not available. Additional studies are needed to clarify the diagnostic value of sensitive TnT and sensitive TnI in terms of mortality and risk stratification in ESRD patients.

On average, patients in this cohort had a TnT value that was 3.5-fold greater than the 99^th^ percentile. Given these elevated baseline values, acute MI can only be diagnosed or ruled out when one or more subsequent samples show a significant rise or fall. Sensitive troponin I offers advantages in this respect, as the plasma concentration of a minority of the patients (14%) was above the 99^th^ percentile.

The study is limited by the paucity of cardiac parameters that were collected, which might have contributed to an unexplained variability of roughly 70% and 50% in plasma troponin I and troponin T concentrations during multivariate modeling. Survival analyses might be limited by the low mortality during the follow-up period. This study focused on nephrological parameters that are commonly available and accessible during hemodialysis treatment, such residual diuresis or shunt flow. Systolic left ventricular function served as a surrogate for cardiac status and showed a high correlation to the plasma troponin concentration. Other cardiac morbidities, such as valvular disease or atrial fibrillation were also contributors of the plasma troponin concentration. Again it should be emphasized that association studies cannot prove causality between the studied parameters and elevated troponin concentrations and cannot distinguish between cause and consequence.

In summary, elevations of plasma troponin concentrations in hemodialysis patients measured with sensitive assays are influenced by cardiac function and dialysis-related parameters. Both are strong predictors of mortality in the short-term and are useful markers to identify patients at risk, however, troponin T is more retained during ESRD compared to troponin I.
